# Association between e-cigarette use and susceptibility to tobacco product use: findings from the 2019 China National Youth Tobacco Survey

**DOI:** 10.3389/fpubh.2023.1272680

**Published:** 2024-01-15

**Authors:** Sixuan Li, Xinying Zeng, Xinbo Di, Shiwei Liu

**Affiliations:** ^1^Ningbo Municipal Center for Disease Control and Prevention, Ningbo, China; ^2^Tobacco Control Office, Chinese Center for Disease Control and Prevention, Beijing, China

**Keywords:** susceptibility, tobacco, e-cigarette, electronic cigarette, adolescent, vaping, nicotine

## Abstract

**Background:**

There is an ongoing debate about whether e-cigarettes act as a gateway to tobacco smoking or contribute to smoking cessation, and relevant studies are limited among Chinese adolescents. This cross-sectional study therefore aimed to explore the relationship between e-cigarette use and susceptibility to tobacco product use among Chinese high school students.

**Methods:**

The study population comprised 107,633 never smokers and 19,377 former smokers, generated from the 2019 China National Youth Tobacco Survey. The primary independent variables of interest were ever e-cigarette use, current e-cigarette use, and the frequency of current e-cigarette use. The main outcome was the susceptibility to tobacco product use. Multilevel logistic regression was used to estimate the association between the primary independent variables of interest and the outcome variable. Moreover, two additional multilevel logistic regression models were fitted using two alternative definitions of the outcome as the sensitivity analyses.

**Results:**

Among never smokers, students who ever used e-cigarettes were more likely to be susceptible to tobacco product use compared to students who never used e-cigarettes (AOR = 2.83, 95%CI = 2.59–3.08). Students who currently used e-cigarettes were more likely to be susceptible to tobacco product use than those who did not currently use e-cigarettes (AOR = 3.89, 95%CI = 3.21–4.72). Among former smokers, with the same settings of modeling, the AORs were 1.76 (95%CI = 1.62–1.91) and 3.16 (95%CI = 2.52–3.97), respectively. Similar results were obtained from the two sensitivity analyses.

**Conclusion:**

Among Chinese high school students, both never smokers and former smokers, e-cigarette use, especially current e-cigarette use, was positively associated with susceptibility to tobacco product use. It is recommended to strengthen the monitoring of e-cigarettes and to provide targeted health education to adolescents.

## 1 Introduction

The tobacco epidemic kills more than 8 million people every year worldwide (1). As the world’s largest producer and consumer of tobacco products, China is facing huge challenges in tobacco control (2). E-cigarettes, known as novel and multi-flavored battery-powered devices, have recently grown in popularity around the world, especially among adolescents and young adults (3, 4).

According to the China Adult Tobacco Survey Report in 2018, the prevalence of past 12-months e-cigarette use and the prevalence of current e-cigarette use aged 15-24 years was 4.4% and 1.5%, respectively. During 2015–2018, the prevalence of current e-cigarette use among those aged 15–24 years increased by 275%, from 0.4 to 1.5% (5). The statistics of the 2019 China National Youth Tobacco Survey (China NYTS) reported that the prevalence of e-cigarette current users among middle and high school students was 2.7 and 3.0%, which were obviously higher than people aged 15–24 years as reported in 2018 (6). However, only 1.2% of middle school students were current e-cigarette smokers in 2014, with a 125% increase from 1.2% in 2014 to 2.7% in 2019(7). For e-cigarette ever users, the prevalence was even higher. In 2019, 10.2% of middle school students and 15.8% of high school students reported that they had ever used e-cigarettes (8).

Most e-cigarettes contain nicotine, which is highly addictive. Children and adolescents who are non-tobacco users might become addicted to nicotine by using e-cigarettes. The experimentation with e-cigarettes may serve as a gateway to future consumption and addiction to tobacco products due to the addictive properties of nicotine (9). Since children and adolescents are in a distinct period of growth and development, they usually have a strong curiosity for novelty and lack awareness of the long-term health risks of tobacco products. Once they start smoking, most of them end up becoming lifelong smokers, which would be detrimental to controlling the tobacco epidemic (7). Meanwhile, a study reported that electronic nicotine delivery systems (ENDS) use could increase the risk of cigarette smoking relapse among former smokers (10).

There is an ongoing debate about whether e-cigarettes act as a gateway to tobacco smoking or whether e-cigarette use contributes to smoking cessation (1, 11, 12). Some studies have concluded that e-cigarette use is not associated with an increased risk of transitioning to daily smoking, and e-cigarettes might serve as a potential smoking cessation tool (13, 14). There is a growing body of studies reporting the longitudinal association between e-cigarette use and the increased risks of subsequent cigarette smoking (15–17). In the World Health Organization (WHO) report on the global tobacco epidemic in 2021, it has been reported that using ENDS could double the risk of smoking cigarettes for children and adolescents. It has been announced that ENDS should be strictly regulated for maximum protection of public health (18). As e-cigarettes become increasingly popular worldwide, many adolescents who have never used tobacco are exposed to nicotine by using e-cigarettes.

Despite the relatively low prevalence of e-cigarette use compared with other countries, there is still a growing concern that the surge in e-cigarette use in recent years in China might undermine the observed decline of tobacco use in the past decades (19). Up to now, no previous study has investigated whether e-cigarette use is associated with susceptibility to tobacco product use among Chinese high school students based on a nationally representative sample. This study aimed to provide relevant evidence for the development of effective tobacco control strategies in China.

## 2 Materials and methods

### 2.1 Study design and participants

From September to December 2019, China NYTS 2019 was conducted, including both middle school students (grades 7th–9th) and high school students (grades 10th–12th). The high school students included both academic high school students and vocational high school students. A three-stage stratified cluster random sampling was used to produce a nationally representative sample while strictly following the sampling manual. In this study, we only selected high school students as the study population. A total of 140,922 students from 974 schools in 31 provinces completed the standardized paper-based questionnaire. The questionnaire consisted of several sections, including basic information (school, grade, class, and individual), cigarette use, e-cigarette use, addiction and cessation, secondhand smoke exposure, tobacco availability and price, tobacco advertisements and promotion, smoking cognition and attitude, and exposure to anti-tobacco media messages. During the academic term, trained investigators distributed the questionnaires to students, who completed them centrally but independently when no teachers were present. The detailed sampling methods and study quality control procedures have been reported in our previous study (6). This study was approved by the China CDC Institutional Review Board (No. 202006), and all information related to participants and schools is kept confidential.

### 2.2 Key measures

#### 2.2.1 Susceptibility to tobacco product use

The main outcome was susceptibility to tobacco product use. Tobacco products are a general term that refers to all tobacco products except e-cigarettes, and in China, mainly conventional cigarettes. Referring to the previous studies (20–23), the following items were used to define the susceptibility to tobacco product use: “If one of your best friends offered you a tobacco product, would you use it?,” and “At any time during the next 12 months do you think you will use any form of tobacco?” Response options included “Definitely not,” “Probably not,” “Probably yes,” and “Definitely yes.” In the basic model, students who reported “Probably not,” “Probably yes,” and “Definitely yes” were categorized as being susceptible to tobacco product use. The participants who chose “definitely not” for both two question items were categorized as not being susceptible to tobacco product use (21). However, it is possible that we underestimated or overestimated the intention due to the specific definition of the outcome. Thus, we fitted two additional regression models using two alternative definitions of susceptibility as the sensitivity analyses. In the first sensitivity analysis, we classified “definitely yes” and “probably yes” as being susceptible to tobacco product use, and participants who chose “probably not” and “definitely not” were categorized as not being susceptible to tobacco product use (20, 21). The participants who chose “definitely not” or “probably not” for both two question items were categorized as not being susceptible to tobacco product use. In the second sensitivity analysis, we used three question items and integrated them into one measure of the susceptibility to tobacco product use. The first two items were the same as the first sensitivity analysis. We then introduced the third question item as follows: “Do you agree or disagree with the following: I think I might enjoy smoking a cigarette.” The response option included “I currently smoke cigarettes,” “strongly agree,” “agree,” “disagree,” and “strongly disagree.” The participants who responded “strongly agree” and “agree” as being susceptible to tobacco product use and “disagree” and “strongly disagree” were categorized as not being susceptible to tobacco product use (23). The participants who did not show susceptibility to tobacco product use for all three question items were categorized as not being susceptible to tobacco product use. The respondents who did not respond to the questions of susceptibility were excluded.

#### 2.2.2 E-cigarette use

The primary independent variables of interest were ever e-cigarette use, current e-cigarette use, and the frequency of current e-cigarette use. The participants who answered “yes” to the question item “Have you ever used e-cigarettes (even only tried once or twice counts)?” were defined as e-cigarette ever users. The question item “During past 30 days, on how many days did you use e-cigarettes?” was used to measure the frequency of current e-cigarette use. The response options included 0, 1–2, 3–5, 6–9, 10–19, 20–29, and 30 days. Participants who reported the frequency of e-cigarette use equal to 1 day or more than 1 day were defined as current e-cigarette users. Additionally, the frequency of current e-cigarette use (0 days/1–9 days/10–30 days) will be used as a continuous variable to estimate the association between the frequency of e-cigarette use and the intention to use tobacco products.

#### 2.2.3 Smoking status

Students who answered “yes” to the question item “Have you ever tried or experimented with cigarette smoking, even one or two puffs?” were defined as ever smokers. Students who answered “no” to the question item “Have you ever tried or experimented with cigarette smoking, even one or two puffs?” were defined as never smokers. Non-current smokers were assessed by the question: “During the past 30 days, on how many days did you smoke cigarettes?” The response options included 0, 1–2, 3–5, 6–9, 10–19, 20–29, and 30 days. If the students chose 0 days, they were defined as non-current smokers. Students who were non-current smokers and ever tried or experimented with smoking were defined as former smokers (smoked but quit).

### 2.3 Statistical analysis

The weighted proportions and prevalence of susceptibility to tobacco product use of the two study samples (never smokers and former smokers) were calculated, accounting for the complex sampling design. The final weight for each individual was obtained by multiplying the sample selection weight, non-response adjustment coefficient, and post-stratification factor (6). The point values and 95% confidence intervals (CIs) were reported. The Rao-Scott chi-square test was used to compare the susceptibility across subgroups.

The multilevel logistic regression was used to estimate the adjusted odds ratios (AORs) that examine the association between the primary independent variables of interest (i.e., e-cigarette ever use, e-cigarette current use, and the frequency of e-cigarette use) and the outcome variable (i.e., susceptibility to tobacco product use). In the present study, a two-level random intercept model was applied to remove the cluster effect of school. Level 2 was considered for school, and Level 1 was the student. The covariate of Level 2 was school type. The covariates of Level 1 were divided into three groups as follows: demographic factors, psychosocial-related factors, and associated tobacco exposure factors (e.g., secondhand smoke exposure and tobacco advertisement exposure). All multilevel logistic models were gradually fitted with three groups of covariates from Level 1. Whether the multilevel regression was appropriate or not was checked by the *p*-value of the intra-class correlation coefficient (ICC) using the PROC NLMIXED command. The multilevel logistic regression was applied if the *p*-value of the ICC was less than 0.05. Two sensitivity analyses were conducted to confirm the robustness of the estimations. *p*-values were two-tailed, and a *p*-value less than 0.05 was considered statistically significant. All analyses were conducted via SAS (version 9.4; SAS Institute, Inc., Cary, North Carolina, USA).

## 3 Results

Among 140,922 high school students, a total of 107,633 never smokers and 19,377 former smokers were selected as the study population (excluding 10,298 current smokers). The response rate of high school students was 94.1% (140,922/149,764). In the basic model, the final analytic sample included 107,605 never smokers (median age 15.9 years) and 19,372 former smokers (median age 16.0 years), after excluding the students who did not respond to the question items for defining the main outcome.

Among 107,605 never smokers (excluding 28 students who did not report susceptibility), 5.5% (95%CI = 5.1–5.8%) of students had ever used e-cigarettes, 0.8% (95%CI = 0.7–0.9%) were current e-cigarettes users, 42.7% (95%CI = 41.2–44.2%) were male students, 69.2% (95%CI = 66.4–72.0%) were academic high school students, 41.7% (95%CI = 38.4–45.0%) lived in urban areas, and 9.0% (95%CI = 8.5–9.5%) were susceptible to tobacco product use (Table 1). Among 19,372 former smokers (excluding 5 students who did not report susceptibility), 35.6% (95%CI = 34.0–37.1%) of students had ever used e-cigarettes, 3.6% (95%CI = 2.9–4.3%) were current e-cigarette users, 71.1% (95%CI = 69.4–72.8%) were male students, 68.1% (95%CI = 64.4–71.7%) were academic high school students, 36.3% (95%CI = 32.7–39.9%) lived in urban areas, and 39.6% (95%CI = 38.2–41.0%) of students were susceptible to tobacco product use (Supplementary Table S1).

**Table 1:** Weighted distribution of demographic characteristics, psychosocial factors, associated tobacco exposure factors, and prevalence of susceptibility to tobacco product use among high school students who were never smokers.

**Variables**		**Weighted proportion among never smokers**	**95%CI**	**Weighted prevalence of susceptibility among never smokers**	**95%CI**	**Rao-Scott chi-squared test *p*** ^***a***^
**Ever use of e-cigarettes**	Yes	5.5	5.1–5.8	28.5	26.3–30.6	<0.0001
	No	94.5	94.2–94.9	7.9	7.5–8.3	
**Current use of e-cigarettes**	Yes	0.8	0.7–0.9	44.1	38.2–50.0	<0.0001
	No	99.2	99.1–99.3	8.8	8.3–9.2	
**Sex**	Male	42.7	41.2–44.2	11.8	11.0–12.5	<0.0001
	Female	57.3	55.8–58.8	7.0	6.6–7.4	
**Residence**	Urban	41.7	38.4–45.0	8.7	7.9–9.5	0.22
	Rural	58.3	55.0–61.6	9.3	8.7–9.9	
**School**	Academic high school	69.2	66.4–72.0	7.9	7.4–8.3	<0.0001
	Vocational high school	30.8	28.0–33.6	11.7	10.5–13.0	
**Grade**	10th	34.6	33.3–35.8	8.1	7.6–8.6	<0.0001
	11th	33.1	32.2–34.0	9.3	8.7–10.0	
	12th	32.4	31.1–33.6	9.7	9.0–10.4	
**Pocket money (RMB)**	0	10.7	10.0–11.4	10.4	9.4–11.5	<0.0001
	≤10	5.9	5.5–6.3	6.5	5.6–7.5	
	11–20	11.6	10.9–12.3	7.6	6.6–8.5	
	21–30	11.3	10.8–11.9	8.7	7.4–9.9	
	31–40	7.3	6.8–7.8	8.2	6.7–9.6	
	41–50	10.9	10.3–11.6	8.4	7.2–9.6	
	>50	42.2	40.7–43.7	9.8	9.3–10.3	
**Think the smoke from other people’s tobacco smoking is harmful**	Definitely not	1.5	1.3–1.7	14.7	12.0–17.5	<0.0001
	Probably not	1.0	0.9–1.1	26.3	21.7–30.8	
	Probably yes	15.9	15.3–16.6	13.0	12.2–13.8	
	Definitely yes	81.7	80.9–82.3	8.0	7.5–8.5	
**Parents smoke tobacco**	None	45.7	44.6–46.9	7.6	7.1–8.2	<0.0001
	Both	1.5	1.3–1.6	14.6	12.0–17.2	
	Father only	52.5	51.3–53.6	9.9	9.3–10.5	
	Mother only	0.3	0.3–0.4	8.1	5.7–10.5	
**Closest friends smoke tobacco**	None of them	55.2	54.0–56.4	4.6	4.2–5.0	<0.0001
	Some of them	41.9	40.8–43.0	13.7	12.9–14.5	
	Most of them	2.4	2.2–2.6	25.2	22.3–28.1	
	All of them	0.4	0.4–0.5	33.8	25.9–41.7	
**Think smoking tobacco makes young people look more or less attractive**	More attractive	4.7	4.3–5.0	22.7	20.3–25.2	<0.0001
	Less attractive	70.4	69.6–71.2	5.9	5.5–6.4	
	No difference from non-smokers	24.9	24.2–25.6	15.2	14.4–16.0	
**Think it would be difficult to quit once someone started smoking tobacco**	Definitely not	7.9	7.4–8.4	12.5	11.2–13.8	<0.0001
	Probably not	15.6	15.1–16.2	13.7	12.6–14.9	
	Probably yes	48.9	48.1–49.7	8.4	7.9–8.9	
	Definitely yes	27.6	26.8–28.3	6.5	6.0–7.1	
**Think smoking tobacco helps people feel more comfortable or less comfortable at social gatherings**	More comfortable	2.6	2.3–2.8	24.1	21.2–27.0	<0.0001
	Less comfortable	89.1	88.5–89.6	7.2	6.8–7.6	
	No difference whether smoking or not	8.4	8.0–8.8	24.1	22.4–25.8	
**How many days has anyone smoked inside your home, in your presence.**	0 days	70.6	69.5–71.8	8.2	7.7–8.8	<0.0001
	1–2 days	13.6	12.9–14.4	10.6	9.8–11.4	
	3–4 days	4.1	3.8–4.4	11.6	10.2–13.0	
	5–6 days	1.9	1.8–2.1	11.4	9.5–13.2	
	7 days	9.7	9.2–10.2	11.1	10.1–12.2	
**How many days has anyone smoked in your presence, inside any enclosed public place, other than your home during the past 7 days.**	0 day	49.8	48.4–51.3	7.2	6.6–7.9	<0.0001
	1–2 days	22.4	21.7–23.1	9.2	8.6–9.8	
	3–4 days	9.5	9.1–9.9	11.3	10.2–12.4	
	5–6 days	3.8	3.6–4.1	13.1	11.4–14.8	
	7 days	14.4	13.6–15.2	12.5	11.5–13.5	
**How many days has anyone smoked in your presence, at any outdoor public place during the past 7 days.**	0 day	48.4	47.0–49.8	7.4	6.7–8.1	<0.0001
	1–2 days	23.3	22.6–24.0	8.8	8.2–9.4	
	3–4 days	9.7	9.3–10.1	11.3	10.3–12.3	
	5–6 days	4.2	3.9–4.4	12.1	10.6–13.6	
	7 days	14.4	13.7–15.1	12.3	11.4–13.2	
**See people smoke inside the school building or outside on school property during the past 30 days.**	Yes	50.8	48.8–52.8	11.4	10.9–12.0	<0.0001
	No	49.2	47.2–51.2	6.6	6.0–7.1	
**Any courses about the bad effects of using tobacco during the past 12 months**	Yes	55.7	54.2–57.2	8.0	7.4–8.6	0.001
	No	44.3	42.8–45.8	9.1	8.5–9.7	
**See or hear any anti-tobacco media messages during the past 30 days.**	Yes	65.0	63.9–66.2	8.5	8.0–9.1	<0.001
	No	35.0	33.8–36.1	9.9	9.3–10.6	
**See any advertisements or videos for tobacco products on the Internet during the past 30 days.**	Yes	23.4	22.7–24.2	12.3	11.5–13.2	<0.0001
	No	76.6	75.8–77.3	8.2	7.7–8.7	
**How often do you see a teacher smoking on campus indoors or outdoors during your school days?**	Every day	10.3	9.3–11.3	13.9	12.7–15.0	<0.0001
	Sometimes	43.3	41.5–45.1	10.6	10.0–11.3	
	Never	46.4	43.9–49.0	6.0	5.3–6.6	

^a^The Rao-Scott chi-square test was used to compare the weighted prevalence of susceptibility across subgroups.

### 3.1 Prevalence of susceptibility to tobacco product use

For never smokers, approximately 7.9% (95%CI = 7.5–8.3%) of the students who never tried an e-cigarette reported susceptibility to tobacco product use, while the weighted prevalence of susceptibility was 28.5% (95%CI = 26.3–30.6%) among students who ever tried an e-cigarette. The weighted prevalence of susceptibility was 44.1% (95%CI = 38.2–50.0%) among current e-cigarette users, compared with 8.8% (95%CI=8.3–9.2%) among those who were not current e-cigarette users. The weighted prevalence of susceptibility varied by demographic, psychosocial, and associated tobacco exposure factors among never smokers. Male students (11.8, 95%CI = 11.0–12.5%) reported relatively higher susceptibility than female students (7.0, 95%CI = 6.6–7.4%). The weighted prevalence of susceptibility was 11.7% (95%CI = 10.5–13.0%) among students in vocational high school, compared with 7.9% (95%CI = 7.4–8.3%) among those in academic high school. The weighted prevalence of susceptibility increased from grade 10th to grade 12th. Students whose parents both used tobacco products (14.6, 95%CI = 12.0–17.2%) had the highest susceptibility to tobacco product use compared with other subgroups. Students who had the most (25.2, 95%CI = 22.3–28.1%) or all (33.8, 95%CI = 25.9–41.7%) of their closest friends smoking tobacco had a relatively higher susceptibility compared with other subgroups. Students who considered smoking tobacco makes young people look more attractive (22.7, 95%CI = 20.3–25.2%) reported higher susceptibility compared with other subgroups. Students who thought it would be definitely (6.5, 95%CI = 6.0–7.1%) or probably difficult (8.4, 95%CI = 7.9–8.9%) to quit smoking reported a lower susceptibility. Students who saw teachers who had smoked on campus during every school day showed the highest susceptibility (13.9, 95%CI = 12.7–15.0%). Students who saw people who had smoked inside the school building or outside on school property (11.4, 95%CI = 10.9–12.0%), or had seen advertisements or videos for tobacco products on the Internet (12.3, 95%CI = 11.5–13.2%) exhibited a relatively higher susceptibility (Table 1).

Overall, former smokers had a higher prevalence of susceptibility to tobacco product use than current smokers. Interestingly, almost 77.9% (95%CI = 71.5–84.4%) of non-current e-cigarette users reported susceptibility to tobacco product use, while only 38.1% (95%CI = 36.8–39.5%) of current e-cigarette users reported the susceptibility. There were no sex and grade differences in the weighted prevalence of susceptibility among former smokers. The pattern of susceptibility among different psychosocial factors and associated tobacco exposure factors in former smokers were similar to those in never smokers. The weighted prevalence of susceptibility to different demographic, psychosocial, and associated tobacco exposure factors among former smokers is shown in Supplementary Table S1.

### 3.2 Association between the primary independent variables of interest and the main outcome

#### 3.2.1 Never smokers

For never smokers, the multilevel logistic regression model, including only the main exposure variable and Level 2 covariate (school type), indicated that susceptibility to tobacco product use was positively associated with the ever use of e-cigarettes (OR = 4.69, 95%CI = 4.41–4.99), current use of e-cigarettes (OR = 7.63, 95%CI = 6.64–8.76), and frequency of current e-cigarette use (OR = 5.19, 95%CI = 4.60–5.87). Table 2 shows the AOR assessing the association between e-cigarette use and susceptibility to tobacco product use after gradually including the demographic factors, psychosocial factors, and associated tobacco exposure factors.

**Table 2:** Multilevel logistic regression on e-cigarette use, frequency of e-cigarette use, and susceptibility to tobacco product use among never smokers^a^.

**Main exposure**		**Model1**		**Model2**		**Model3**		**Model4**	
		**OR**	**95%CI**	**AOR**	**95%CI**	**AOR**	**95%CI**	**AOR**	**95%CI**
**Ever use of e-cigarettes**	Yes	4.69	4.41–4.99	4.12	3.87–4.40	2.72	2.54–2.91	2.83	2.59–3.08
	No								
**Current use of e-cigarettes**	Yes	7.63	6.64–8.76	6.57	5.71–7.57	3.85	3.30–4.48	3.89	3.21–4.72
	No								
**Frequency of e-cigarette use**		5.19	4.60–5.87	4.58	4.05–5.18	2.95	2.59–3.37	3.07	2.59–3.63

^a^The population included in the model was 107,605 excluding 28 students who did not report susceptibility. ICC: 0.0516 (*P*-value < 0.0001). Model1: Only include main exposure and school type (level2 variable). Model2: Analyses adjusted for demographic factors. Model3: Analyses adjusted for demographic factors and psychosocial factors. Model4: Analyses adjusted for demographic factors, psychosocial factors, and associated tobacco exposure factors.

**Table 3:** Multilevel logistic regression on e-cigarette use, frequency of e-cigarette use, and susceptibility to tobacco product use among never smokers (sensitivity analysis 1)^a^.

**Main exposure**		**Model1**		**Model2**		**Model3**		**Model4**	
		**OR**	**95%CI**	**AOR**	**95%CI**	**AOR**	**95%CI**	**AOR**	**95%CI**
**Ever use of e-cigarettes**	Yes	5.86	5.41–6.35	5.06	4.66–5.5	3.01	2.76–3.29	3.02	2.71–3.38
	No								
**Current use of e-cigarettes**	Yes	9.15	7.82–10.71	7.62	6.49–8.95	3.91	3.28–4.65	3.76	3.01–4.68
	No								
**Frequency of e-cigarette use**		5.59	4.9–6.37	4.83	4.23–5.51	2.86	2.47–3.3	2.78	2.31–3.35

^a^The population included in the model was 107,602 excluding 31 students who did not report susceptibility. ICC: 0.0629 (*P*-value < 0.0001). Model1: Only include main exposure and school type (level2 variable). Model2: Analyses adjusted for demographic factors. Model3: Analyses adjusted for demographic factors and psychosocial factors. Model4: Analyses adjusted for demographic factors, psychosocial factors, and associated tobacco exposure factors.

After adjusting for all potential covariates, students who ever used e-cigarettes were more likely to be susceptible to tobacco product use than students who had never used e-cigarettes (AOR = 2.83, 95%CI = 2.59–3.08). The students who currently use e-cigarettes were more likely to be susceptible to tobacco product use than students who do not currently use e-cigarettes (AOR = 3.89, 95%CI = 3.21–4.72). The frequency of current e-cigarette use (0 days/1–9 days/10–30 days) increased for each unit, and the odds of susceptibility increased by 3.07 (95%CI = 2.59–3.63). Two sensitivity analyses using two additional definitions of the outcome showed similar results to Table 2 (as shown in Tables 3–4)”. In the first sensitivity analysis, ever use (AOR = 3.02, 95%CI = 2.71–3.38) and current use of e-cigarettes (AOR = 3.76, 95%CI = 3.01–4.68) were associated with susceptibility to tobacco product use after adjusting all the potential covariates. In the second sensitivity analysis, ever use (AOR = 2.77, 95%CI: 2.51–3.06) and current use of e-cigarettes (AOR = 3.22, 95%CI = 2.61–3.97) were associated with susceptibility to tobacco product use after adjusting all the potential covariates.

**Table 4:** Multilevel logistic regression on e-cigarette use, frequency of e-cigarette use, and susceptibility to tobacco product use among never smokers (sensitivity analysis 2)^a^.

**Main exposure**		**Model1**		**Model2**		**Model3**		**Model4**	
		**OR**	**95%CI**	**AOR**	**95%CI**	**AOR**	**95%CI**	**AOR**	**95%CI**
**Ever use of e-cigarettes**	Yes	5.26	4.89–5.65	4.52	4.20–4.86	2.86	2.64–3.10	2.77	2.51–3.06
	No								
**Current use of e-cigarettes**	Yes	7.91	6.82–9.18	6.61	5.68–7.68	3.51	2.97–4.14	3.22	2.61–3.97
	No								
**Frequency of e-cigarette use**		5.45	4.80–6.19	4.71	4.14–5.35	2.81	2.44–3.23	2.58	2.16–3.09

^a^The population included in the model was 106,866 excluding 767 students who did not report susceptibility. ICC: 0.0457 (P-value < 0.0001). Model1: Only include main exposure and school type (level2 variable). Model2: Analyses adjusted for demographic factors. Model3: Analyses adjusted for demographic factors and psychosocial factors. Model4: Analyses adjusted for demographic factors, psychosocial factors, and associated tobacco exposure factors.

#### 3.2.2 Former smokers

For former smokers, the multilevel logistic regression model, including only the main exposure variable and Level 2 covariate (school type), indicated that susceptibility to tobacco product use was positively associated with the ever use of e-cigarettes (OR = 2.12, 95%CI = 2.00–2.26), current use of e-cigarettes (OR = 3.96, 95%CI = 3.34–4.70), and frequency of current e-cigarette use (OR = 3.21, 95%CI = 2.76–3.72). Supplementary Table S2 shows the AOR assessing the association between e-cigarette use and susceptibility to tobacco product use after gradually including the demographic factors, psychosocial factors, and associated tobacco exposure factors.

After adjusting for all potential covariates, students who ever used e-cigarettes were more likely to be susceptible to tobacco product use than students who had never used e-cigarettes (AOR = 1.76, 95%CI = 1.62–1.91); students who currently used e-cigarettes were more likely to be susceptible to tobacco product use than students who did not currently use e-cigarettes (AOR = 3.16, 95%CI = 2.52–3.97). The frequency of current e-cigarette use (0 days/1–9 days/10–30 days) increased for each unit, and the odds of susceptibility increased by 2.69 (95%CI = 2.21–3.27). Two sensitivity analyses using two additional definitions of the outcome showed similar results to Supplementary Table S2 (as shown in Supplementary Table S3–S4). In the first sensitivity analysis, ever use (AOR = 1.69, 95%CI = 1.54–1.85) and current use of e-cigarettes (AOR = 2.95, 95%CI = 2.40–3.64) were associated with susceptibility to tobacco product use after adjusting all the potential covariates. In the second sensitivity analysis, ever use (AOR = 1.65, 95%CI=1.51–1.80) and current use of e-cigarettes (AOR = 2.79, 95%CI = 2.25–3.45) were associated with susceptibility to tobacco product use after adjusting all the potential covariates.

## 4 Discussion

To the best of our knowledge, the present study was the first to investigate whether the use of e-cigarettes is associated with susceptibility to tobacco product use among Chinese high school students using a nationally representative sample. Our findings indicated that the use of e-cigarettes was positively associated with susceptibility to tobacco product use among both never and former smokers after adjusting for demographic, psychosocial, and associated tobacco exposure factors. Among never smokers, the students who ever used e-cigarettes had 2.83 times higher odds of susceptibility than students who had never used e-cigarettes, and the students who currently use e-cigarettes had 3.89 times higher odds of susceptibility than students who do not currently use e-cigarettes. Meanwhile, there was a dose–response relationship between the frequency of e-cigarette use and susceptibility to tobacco product use. Moreover, we used two additional definitions of susceptibility to tobacco product use as the sensitivity analyses, and the positive association was still significant.

Our findings were consistent with previous studies. A cross-sectional study among never-smoking US students concluded that ever e-cigarette users had 1.7 times higher adjusted odds for having smoking intentions than never users (21). This study used the same definition of susceptibility as ours but included both middle and high school students. A Canadian cross-sectional study among grades 10th–12th students reported that students who had ever tried e-cigarette had 1.98 times higher odds of smoking susceptibility compared to non-ever users, and current users of e-cigarettes had 2.48 times higher odds of smoking susceptibility compared to non-current users (24). This study assessed susceptibility to smoking following three questions based on the Pierce et al. algorithm, which includes an additional question (i.e., Do you think you will try a cigarette soon?) compared to our study (25). A previous study conducted among Chinese middle school students explored the association between e-cigarette use and susceptibility to tobacco use using three questions among never smokers. However, in this study, these three question items were not integrated into one measure of susceptibility but assessed separately based on each question item. This study reported that e-cigarette users had 6.97 times higher odds of intention to use a tobacco product in the next 12 months, 5.14 times higher odds of using a tobacco product if a best friend offered tobacco products to them, and 14.63 times higher odds of being more likely to say they might enjoy smoking a cigarette compared with non-e-cigarette users (23). The recent systematic review found that ever use of ENDS or Electronic Non-Nicotine Delivery Systems (ENNDS) was more than twice as likely to later use conventional cigarettes among children and adolescents aged<20 years, which provided strong evidence to support the causal relationship between ever ENDS/ENNDS use and later smoking for youths (26). The AORs of our study were relatively higher than the studies conducted in the US and Canada and the AORs reported on the report of WHO (18) might be due to the different definitions of susceptibility, ethnicity, population included, analysis techniques, and survey time. However, all these studies indicated a positive correlation between e-cigarette use and susceptibility to tobacco product use.

Among US high school students, e-cigarette use has increased dramatically, with the prevalence of current e-cigarette use rising from 1.5% in 2011 to 19.6% in 2020 (19, 27). In China, the prevalence of e-cigarette use remains low but has increased substantially (7, 8). Since e-cigarette use among youths is positively associated with susceptibility to tobacco product use, mainly conventional cigarettes in China, it is crucial to counteract the rising trend of e-cigarette use among youths. China has issued a series of policies to regulate the e-cigarette market since 2018, and the online ban on the sale of e-cigarettes was implemented in 2019 (28). In order to strengthen the supervision of e-cigarettes in China, e-cigarettes have been regulated in the same way as cigarettes, according to China’s Tobacco Monopoly Law since 10 November 2021. A cross-sectional study conducted in six provincial-level administrative divisions among Chinese secondary school students in 2021 found that 67% of current e-cigarette users were able to purchase e-cigarettes without age restrictions, and 36.3% of current e-cigarette users bought e-cigarettes online. These results indicated inadequate enforcement of e-cigarette policies (29).

Some researchers in the US believed that the recent changes in patterns of e-cigarettes and other tobacco products offset the decline in tobacco product use that occurred in previous years (30). For China, the increased consumption of e-cigarettes might hinder the achievement of the Health China 2030 target of reducing the smoking rate to 20% by 2030 (31). Hence, there is an urgent need to take action to prevent youths from becoming addicted to nicotine throughout their lives by using e-cigarettes. Comprehensive policies are needed to regulate the availability, accessibility, and marketing of e-cigarettes to children and adolescents in China. Given the current situation in China, we recommend that e-cigarette offline stores be banned in crowded places; meanwhile, more health education and promotion campaigns are required to raise awareness of the harmful health effects of e-cigarettes among youths. Moreover, tobacco monopoly administrative departments and market supervision departments should strengthen the online supervision of e-cigarettes. For students who are willing to quit e-cigarettes, we recommend that schools provide appropriate help on the premise of protecting students’ privacy.

In our study, there are some limitations. First, as a cross-sectional study, it is impossible to establish the causal or temporal direction of the association reported in the study. However, either scenario has important public health implications. Second, since it is a self-reported questionnaire, recall bias and reporting bias might occur to some degree. Third, the questionnaire does not include items related to personality characteristics, which might be potential confounders. It was reported that personality characteristics such as rebelliousness, risk-taking, depression, and anxiety were risk factors for smoking in childhood or adolescence (32). However, we gradually included all the potential confounders that we collected in the questionnaire in the regression model and used two sensitivity analyses to confirm the robustness of the positive association between e-cigarette use and susceptibility to tobacco product use. While longitudinal studies were needed to further explore the casual relationship, more information can be collected in the questionnaire, including the students’ personality traits, the brands and flavors of e-cigarettes, social responsiveness, and relatedness (24).

In conclusion, among Chinese high school students, both never smokers and former smokers, e-cigarette use, especially current e-cigarette use, was positively associated with susceptibility to tobacco product use. It is recommended to strengthen the monitoring of e-cigarettes and to provide targeted health education to adolescents.

## Data availability statement

The datasets presented in this article are not readily available because individual participant data in our study will not be made available publicly. Requests to access the datasets should be directed to for further detailed data access policy and procedure, please contact chinatco@126.com.

## Ethics statement

The studies involving humans were approved by China CDC Institutional Review Board (No. 202006). The studies were conducted in accordance with the local legislation and institutional requirements. Written informed consent for participation was not required from the participants or the participants’ legal guardians/next of kin because School officials and respondents provided oral consent to participate before the interview.

## Author contributions

SXL: Conceptualization, Formal analysis, Methodology, Writing – original draft. XZ: Data curation, Methodology, Writing – original draft. XD: Formal analysis, Investigation, Writing – original draft. SWL: Data curation, Supervision, Writing – review & editing.

## References

[1] Chan GCK Stjepanović D Lim C Sun T Shanmuga Anandan A Connor JP. Gateway or common liability? A systematic review and meta-analysis of studies of adolescent e-cigarette use and future smoking initiation. Addiction. (2020) 116:743–56. doi: 10.1111/add.15246 32888234

[2] Fang J Yang G Wan X. “Pro-tobacco propaganda”: a case study of tobacco industry-sponsored elementary schools in China. Tob Control. (2020) 29:447–51. doi: 10.1136/tobaccocontrol-2018-054646, PMID: 31302606 PMC7361027

[3] Cullen KA Gentzke AS Sawdey MD Chang JT Anic GM Wang TW. E-cigarette use among youth in the United States, 2019. JAMA. (2019) 322:2095–103. doi: 10.1001/jama.2019.18387, PMID: 31688912 PMC6865299

[4] Zhao Z Zhang M Wu J Xu X Yin P Huang Z. E-cigarette use among adults in China: findings from repeated cross-sectional surveys in 2015–16 and 2018–19. Lancet Public Heal. (2020) 5:e639–49. doi: 10.1016/S2468-2667(20)30145-6, PMID: 33271077

[5] Chinese Center for Disease Control and Prevention In: China adult tobacco survey report in (2018) Beijing: People’s Medical Publishing House (2020)

[6] Liu S Xiao L Zeng X. Tobacco use and exposure among secondary school students — China, 2019. China CDC Wkly. (2020) 2:385–93. doi: 10.46234/ccdcw2020.099

[7] Xiaofeng L. China youth tobacco survey report in 2014. Beijing: People’s Medical Publishing House (2015).

[8] Lin X Xinbo D Shiwei L Xinying Z Xinhua LI. Current status of e-cigarette use among Chinese secondary school students. In: The 21st National Symposium on Tobacco Control and the 30th Anniversary of the Establishment of The Chinese Tobacco Control Association. Chinese Association on Tobacco Control (2020) 31–32.

[9] Kinnunen JM Ollila H Minkkinen J Lindfors PL Timberlake DS Rimpelä AH. Nicotine matters in predicting subsequent smoking after e-cigarette experimentation: a longitudinal study among Finnish adolescents. Drug Alcohol Depend. (2019) 201:182–7. doi: 10.1016/j.drugalcdep.2019.04.019, PMID: 31238240

[10] Everard CD Silveira ML Kimmel HL Marshall D Blanco C Compton WM. Association of Electronic Nicotine Delivery System use with Cigarette Smoking Relapse among Former Smokers in the United States. JAMA Netw Open. (2020) 3:e204813. doi: 10.1001/jamanetworkopen.2020.4813, PMID: 32501492 PMC7275247

[11] Etter JF. Gateway effects and electronic cigarettes. Addiction. (2018) 113:1776–83. doi: 10.1111/add.13924 28786147

[12] Khouja JN Suddell SF Peters SE Taylor AE Munafò MR. Is e-cigarette use in non-smoking young adults associated with later smoking? A systematic review and meta-analysis. Tob Control. (2021) 30:8–15. doi: 10.1136/tobaccocontrol-2019-055433, PMID: 32156694 PMC7803902

[13] Chyderiotis S Benmarhnia T Beck F Spilka S Legleye S. Does e-cigarette experimentation increase the transition to daily smoking among young ever-smokers in France? Drug Alcohol Depend. (2019) 208:107853. doi: 10.1016/j.drugalcdep.2020.107853, PMID: 31958678

[14] Grabovac I Oberndorfer M Fischer J Wiesinger W Haider S Dorner TE. Effectiveness of electronic cigarettes in smoking cessation: a systematic review and meta-analysis. Nicotine Tob Res. (2020) 23:625–34. doi: 10.1093/ntr/ntaa181 32939543

[15] Leventhal AM Strong DR Kirkpatrick MG Unger JB Sussman S Riggs NR. Association of electronic cigarette use with initiation of combustible tobacco product smoking in early adolescence. JAMA. (2015) 314:700–7. doi: 10.1001/jama.2015.8950, PMID: 26284721 PMC4771179

[16] Pierce JP Chen R Leas EC White MM Kealey S Stone MD. Use of E-cigarettes and other tobacco products and progression to daily cigarette smoking. Pediatrics. (2021) 147:e2020025122. doi: 10.1542/peds.2020-025122, PMID: 33431589 PMC7849197

[17] Barrington-Trimis JL Urman R Berhane K Unger JB Cruz TB Pentz MA. E-cigarettes and future cigarette use. Pediatrics. (2016) 138:1. doi: 10.1542/peds.2016-0379, PMID: 27296866 PMC4925085

[18] World Health Organization. WHO report on the global tobacco epidemic 2021: addressing new and emerging products (2021). Available at: https://www.who.int/news-room/q-a-detail/tobacco-e-cigarettes (Accessed August 29, 2021)

[19] Wang TW Neff LJ Park-Lee E Ren C Cullen KA King BA. E-cigarette use among middle and high school students — United States, 2020. MMWR Morb Mortal Wkly Rep. (2020) 69:1310–2. doi: 10.15585/mmwr.mm6937e1, PMID: 32941408 PMC7498174

[20] Wang L Chen J Ho SY Leung LT Wang MP Lam TH. Exposure to e-cigarette advertising, attitudes, and use susceptibility in adolescents who had never used e-cigarettes or cigarettes. BMC Public Health. (2020) 20:1349. doi: 10.1186/s12889-020-09422-w, PMID: 32887586 PMC7650221

[21] Bunnell RE Agaku IT Arrazola RA Apelberg BJ Caraballo RS Corey CG. Intentions to smoke cigarettes among never-smoking US middle and high school electronic cigarette users: national youth tobacco survey, 2011-2013. Nicotine Tob Res. (2015) 17:228–35. doi: 10.1093/ntr/ntu166, PMID: 25143298 PMC4515756

[22] Coleman BN Apelberg BJ Ambrose BK Green KM Choiniere CJ Bunnell R. Association between electronic cigarette use and openness to cigarette smoking among US young adults. Nicotine Tob Res. (2015) 17:212–8. doi: 10.1093/ntr/ntu211, PMID: 25378683 PMC4892708

[23] Xiao L Parascandola M Wang C Jiang Y. Perception and current use of E-cigarettes among youth in China. Nicotine Tob Res. (2019) 21:1401–7. doi: 10.1093/ntr/nty145, PMID: 30053201 PMC6941706

[24] Azagba S Baskerville NB Foley K. Susceptibility to cigarette smoking among middle and high school e-cigarette users in Canada. Prev Med (Baltim). (2017) 103:14–9. doi: 10.1016/j.ypmed.2017.07.017, PMID: 28735725

[25] Pierce JP Choi WS Gilpin EA Farkas AJ Merritt RK. Validation of usceptibility as a predictor of which adolescents take up smoking in the United States. Health Psychol. (1996) 15:355–61. doi: 10.1037/0278-6133.15.5.355, PMID: 8891714

[26] Yoong SL Hall A Turon H Stockings E Leonard A Grady A. Association between electronic nicotine delivery systems and electronic non-nicotine delivery systems with initiation of tobacco use in individuals aged < 20 years. A systematic review and meta-analysis. PLoS One. (2021) 16:e0256044. doi: 10.1371/journal.pone.0256044, PMID: 34495974 PMC8425526

[27] Cullen KA Liu ST Bernat JK Slavit WI Tynan MA King BA. Morbidity and mortality weekly report flavored tobacco product use among middle and high school students-United States, 2014-2018. (2019). Available at: https://www.cdc.gov/tobacco/data_statistics/surveys/nyts/index.htm (Accessed August 8, 2021) 10.15585/mmwr.mm6839a2 PMC6776376 31581163

[28] Notice on further protecting minors from electronic cigarettes. State Administration for Market Regulation and State Tobacco Monopoly Administration. (2019) Available at: http://www.gov.cn/xinwen/2019-11/01/content_5447612.htm (Accessed February 7, 2021)

[29] Qi Z Lin B Xie X Xiao L. Characteristics and associated factors of E-cigarette use among secondary school students — 6 PLADs in China (2022) China CDC Wkly. 4:635–9. doi: 10.46234/ccdcw2022.126 PMC9339352 35919825

[30] Cullen KA Bernat JK Slavit WI Tynan MA King BA Liu ST. Morbidity and mortality weekly report flavored tobacco product use among middle and high school students-United States, 2014-2018. MMWR Morb Mortal Wkly Rep. (2019) 68:839–44. doi: 10.15585/mmwr.mm6839a2, PMID: 31581163 PMC6776376

[31] Goodchild M Zheng R. Tobacco control and healthy China 2030. Tob Control. (2019) 28:409–13. doi: 10.1136/tobaccocontrol-2018-054372, PMID: 30030408 PMC6589446

[32] Benowitz NL. Nicotine addiction. N Engl J Med. (2010) 362:2295. doi: 10.1056/NEJMRA0809890 20554984 PMC2928221

