# Supplementary material for: Association between e-cigarette use and susceptibility to tobacco product use: findings from the 2019 China National Youth Tobacco Survey
Source: Front Public Health. 2024 Jan 15;11:1272680. doi: 10.3389/fpubh.2023.1272680 (PMC10823011; doi:10.3389/fpubh.2023.1272680)
Supplement: Supplementary file 1 [file Data_Sheet_1.pdf]

eTable1 Weighted distribution of demographic characteristics, psychosocial factors, associated tobacco exposure factors and prevalence of susceptibility to tobacco product use among high school students who were former smokers

**Variables**		**Weighted Proportion among former smokers**	**95%CI**	**Weighted Prevalence of susceptibility among former smokers**	**95%CI**	**Rao-Scott Chi-Squared test *p*[a]**
**Ever use of e-cigarettes**	Yes	35.6	34.0-37.1	51.0	48.7-53.3	<0.0001
	No	64.4	62.9-66.0	33.2	31.6-34.8	
**Current use of e-cigarettes**	Yes	3.6	2.9-4.3	38.1	36.8-39.5	<0.0001
	No	96.4	95.7-97.1	77.9	71.5-84.4	
**Sex**	Male	71.1	69.4-72.8	39.9	38.3-41.6	0.402
	Female	28.9	27.2-30.6	38.7	36.1-41.3	
**Residence**	Urban	36.3	32.7-39.9	41.1	38.8-43.4	0.103
	Rural	63.7	60.1-67.3	38.7	37.0-40.5	
**School**	Academic high school	68.1	64.4-71.7	37.5	36.1-38.9	<0.0001
	Vocational high school	31.9	28.3-35.6	44.0	41.2-46.8	
**Grade**	10th	32.4	31.1-33.7	38.8	36.5-41.0	0.528
	11th	32.4	30.9-33.9	39.5	37.5-41.5	
	12th	35.2	33.3-37.1	40.3	38.2-42.5	
**Pocket money (RMB)**	0	9.2	8.5-10.0	40.9	37.3-44.6	0.002
	≤10	4.9	4.1-5.6	32.4	26.1-38.6	
	10-20	9.4	8.2-10.6	37.5	33.9-41.0	
	21-30	11.0	10.2-11.8	36.4	33.0-39.8	
	31-40	7.1	6.5-7.7	40.1	36.0-44.2	
	41-50	9.9	9.2-10.7	37.8	34.9-40.8	
	>50	48.4	46.2-50.6	41.6	39.8-43.3	
**Think the smoke from other people's tobacco smoking is harmful**	Definitely not	1.4	1.1-1.6	36.3	28.9-43.7	<0.0001
	Probably not	1.5	1.0-1.9	70.8	60.2-81.5	
	Probably yes	15.8	14.9-16.7	52.2	49.1-55.2	

	Definitely yes	81.4	80.5-82.3	36.6	35.3-38.0	
**Parents smoke tobacco**	None	38.1	36.5-39.7	37.9	35.7-40.2	0.171
	Both	2.4	2.1-2.7	40.1	33.6-46.6	
	Father only	59.0	57.4-60.5	40.4	38.8-41.9	
	Mother only	0.5	0.3-0.6	42.2	28.0-56.4	
**Closest friends smoke tobacco**	None of them	20.0	18.9-21.1	19.4	17.4-21.3	<0.0001
	Some of them	70.3	69.4-71.3	43.3	41.7-44.9	
	Most of them	8.7	7.8-9.6	53.3	49.0-57.6	
	All of them	1.0	0.8-1.2	63.4	52.7-74.0	
**Think smoking tobacco makes young people look more or less attractive**	More attractive	7.6	7.0-8.3	53.8	50.1-57.4	<0.0001
	Less attractive	53.3	52.0-54.7	30.8	28.9-32.7	
	No difference from non-smokers	39.0	37.7-40.4	48.7	46.5-50.9	
**Think it would be difficult to quit once someone started smoking tobacco**	Definitely not	12.8	12.0-13.6	43.4	40.9-45.9	<0.0001
	Probably not	23.6	22.6-24.6	47.0	44.8-49.1	
	Probably yes	44.8	43.8-45.8	37.3	35.2-39.4	
	Definitely yes	18.8	17.7-19.8	33.1	30.5-35.7	
**Think smoking tobacco helps people feel more comfortable or less comfortable at social gatherings**	More comfortable	5.1	4.6-5.6	58.5	53.4-63.7	<0.0001
	Less comfortable	77.6	76.5-78.6	32.7	31.1-34.4	
	No difference whether smoking or not	17.3	16.5-18.2	64.6	62.1-67.1	
**How many days has anyone smoked inside your home, in your presence.**	0 day	59.2	57.5-60.9	38.8	37.0-40.5	0.043
	1 to 2 days	13.8	12.9-14.7	37.6	34.6-40.7	
	3 to 4 days	5.8	5.3-6.4	44.2	38.7-49.6	
	5 to 6 days	3.2	2.7-3.7	42.5	36.6-48.5	
	7 days	17.9	16.9-18.9	41.8	39.0-44.5	
**How many days has anyone smoked in your presence, inside any enclosed public place, other than your home during the past 7 days.**	0 day	29.2	28.0-30.5	36.6	34.0-39.2	<0.001
	1 to 2 days	20.7	19.7-21.7	37.3	34.9-39.6	
	3 to 4 days	12.5	11.7-13.3	40.1	37.1-43.1	
	5 to 6 days	7.1	6.5-7.7	43.1	38.2-47.9	
	7 days	30.3	29.1-31.8	43.1	40.9-45.2	

**How many days has anyone smoked in your presence, at any outdoor public place during the past 7 days.**	0 day	30.5	29.4-31.7	35.7	33.6-37.7	<0.0001
	1 to 2 days	22.2	21.2-23.3	35.3	32.9-37.8	
	3 to 4 days	12.9	12.2-13.7	43.2	39.7-46.7	
	5 to 6 days	6.7	6.0-7.5	42.6	37.6-47.5	
	7 days	27.5	26.4-28.7	44.9	42.9-46.9	
**See people smoke inside the school building or outside on school property during the past 30 days.**	Yes	76.2	74.6-77.7	41.0	39.3-42.7	<0.001
	No	23.8	22.3-25.4	35.0	32.5-37.6	
**Any courses about the bad effects of using tobacco during the past 12 months**	Yes	47.1	45.2-49.0	39.1	37.8-40.4	0.903
	No	52.9	51.0-54.8	38.9	36.6-41.2	
**See or hear any anti-tobacco media messages during the past 30 days.**	Yes	55.2	54.0-56.5	39.7	38.0-41.5	0.707
	No	44.8	43.5-46.0	39.4	37.6-41.1	
**See any advertisements or videos for tobacco products on the Internet during the past 30 days.**	Yes	23.0	21.5-24.4	46.4	44.0-48.8	<0.0001
	No	77.0	75.6-78.5	38.3	36.8-39.9	
**How often do you see a teacher smoking on campus indoors or outdoors during your school days?**	Every day	17.9	16.2-19.5	46.2	43.4-49.1	<0.0001
	Sometimes	54.0	52.4-55.7	40.1	38.2-42.0	
	Never	28.1	25.9-30.3	34.3	31.9-36.8	

[a] The Rao-Scott chi square test was used to compare the weighted prevalence of susceptibility across subgroups.

eTable2 Multilevel logistic regression on e-cigarette use, frequency of e-cigarette use and susceptibility to tobacco product use among former smokers[a]

**Main Exposure**		**Model1**		**Model2**		**Model3**		**Model4**	
		**OR**	**95%CI**	**AOR**	**95%CI**	**AOR**	**95%CI**	**AOR**	**95%CI**
**Ever use of e-cigarettes**	Yes	2.12	2.00-2.26	2.20	2.07-2.35	1.78	1.66-1.91	1.76	1.62-1.91
	No								
**Current use of e-cigarettes**	Yes	3.96	3.34-4.70	3.99	3.36-4.74	3.12	2.6-3.74	3.16	2.52-3.97
	No								
**Frequency of e-cigarette use**		3.21	2.76-3.72	3.22	2.77-3.75	2.63	2.25-3.08	2.69	2.21-3.27

[a]The population included in the model was 19372 excluding 5 students who did not report susceptibility.

ICC: 0.0353 (P value < 0.0001).

Model1: Only include main exposure and school type (level2 variable)

Model2: Analyses adjusted for demographic factors

Model3: Analyses adjusted for demographic factors and psychosocial factors.

Model4: Analyses adjusted for demographic factors, psychosocial factors and associated tobacco exposure factors.

eTable3 Multilevel logistic regression on e-cigarette use, frequency of e-cigarette use and susceptibility to tobacco product use among former smokers (sensitivity analysis 1)[a]

Main Exposure		Model1		Model2		Model3		Model4	
		OR	95%CI	AOR	95%CI	AOR	95%CI	AOR	95%CI
**Ever use of e-cigarettes**	Yes	2.18	2.04-2.34	2.26	2.10-2.42	1.75	1.62-1.89	1.69	1.54-1.85
	No								
**Current use of e-cigarettes**	Yes	3.79	3.24-4.43	3.81	3.25-4.46	2.88	2.43-3.41	2.95	2.40-3.64
	No								
**Frequency of e-cigarette use**		3.06	2.68-3.50	3.07	2.69-3.51	2.48	2.15-2.86	2.56	2.15-3.06

[a]The population included in the model was 19371 excluding 6 students who did not report susceptibility.

ICC: 0.0253 (P value < 0.0001).

Model1: Only include main exposure and school type (level2 variable)

Model2: Analyses adjusted for demographic factors

Model3: Analyses adjusted for demographic factors and psychosocial factors.

Model4: Analyses adjusted for demographic factors, psychosocial factors and associated tobacco exposure factors.

eTable4 Multilevel logistic regression on e-cigarette use, frequency of e-cigarette use and susceptibility to tobacco product use among former smokers (sensitivity analysis 2)[a]

**Main Exposure**		**Model1**		**Model2**		**Model3**		**Model4**	
		**OR**	**95%CI**	**AOR**	**95%CI**	**AOR**	**95%CI**	**AOR**	**95%CI**
**Ever use of e-cigarettes**	Yes	2.16	2.02-2.30	2.21	2.07-2.37	1.74	1.61-1.87	1.65	1.51-1.80
	No								
**Current use of e-cigarettes**	Yes	3.72	3.18-4.36	3.7	3.16-4.35	2.74	2.31-3.27	2.79	2.25-3.45
	No								
**Frequency of e-cigarette use**		3.04	2.66-3.49	3.03	2.64-3.48	2.39	2.06-2.76	2.42	2.02-2.89

[a]The population included in the model was 19,177 excluding 200 students who did not report susceptibility.

ICC: 0.0259 (P value < 0.0001).

Model1: Only include main exposure and school type (level2 variable)

Model2: Analyses adjusted for demographic factors

Model3: Analyses adjusted for demographic factors and psychosocial factors.

Model4: Analyses adjusted for demographic factors, psychosocial factors and associated tobacco exposure factors.
